# Editorial: Soft computing and artificial intelligence techniques in decision making, management and engineering

**DOI:** 10.3389/frai.2026.1954913

**Published:** 2026-09-08

**Authors:** Ernesto Leon-Castro, Jose M. Merigo, Fabio Blanco-Mesa, Victor G. Alfaro Garcia

**Affiliations:** 1 Universidad Catolica de la Santisima Concepcion, Concepción, Chile; 2 University of Technology Sydney, Sydney, NSW, Australia; 3 Universidad Pedagógica y Tecnológica de Colombia, Tunja, Colombia; 4 Universidad Michoacana de San Nicolas de Hidalgo, Morelia, Mexico

**Keywords:** artificial intelligence, decision-making, engineering, management, soft computing

The evolution of Soft Computing and Artificial Intelligence has increased significantly in recent decades (Aqil Burney et al., 2017; Xiaolong et al., 2021). The methodologies and techniques within the area have enabled advances in the improvement processes of different areas (Akeiber, 2025), generating better decision-making (Blanco-Mesa et al., 2017), with administration and engineering among the most relevant (Al-Khamees et al., 2026). In this sense, the Research Topic entitled *Soft computing and artificial intelligence techniques in decision making, management and engineering* is a compilation of 7 articles which allow not only to identify the techniques, but also to visualize their application to real problems within the area, which will allow the reader to know much more about the processes that must be followed for a good practical functioning of these tools.

Within this Research Topic, articles are developed that solve problems in different areas. Within the Human Resources area, the article “*Selecting self-and-follower goal-aware leadership styles across sectors: a decision support approach*” (Czukor et al.) presents an artificial intelligence, fuzzy logic, and machine learning model used to solve the problem of evaluating and selecting the optimal leader in different industries. This application is relevant since it can be identified that each of the industries has different needs, so the evaluation will depend on what is being sought, such as occupational safety, innovation, or employee commitment, so it is identified that it is not a one-solution problem. This not only allows us to identify the importance of Multi-criteria Decision-Making (MCDM) techniques in this topic, but also provides a new theoretical vision toward leadership studies.

Regarding the accounting area, the work “*Minimizing unnecessary tax audits using multi-objective hyperparameter tuning of XGBoost with focal loss*” (Malashin et al.) is presented, which identifies how machine learning models can be used for audit processes and the detection of anomalies in tax data. By using Non-dominated Sorting Genetic Algorithm II (NSGA-II) to optimize the XGBoost model, it was possible to obtain better results for identifying discrepancies in records and reports, which would allow greater transparency and reduce the workload for internal audits within organizations.

Within the Research Topic, you can find two articles that discuss customer retention using different techniques and sectors. The first is “*A predictive analytics approach to improve telecom's customer retention*” (Omari et al.) which addresses a model that uses various prediction models such as Support Vector Machines (SVM), Logistic Regression, K-Nearest Neighbors (KNN), and Naive Bayes to improve customer retention strategies, identifying different strategies based on the current situation of each one and allowing to identify when high-value customers are most likely to leave, which allows the company to generate retention activities and can reduce the costs associated with looking for new customers. The second is “*The role of AI-enhanced fast delivery services in strengthening customer retention and loyalty in competitive markets*” (Kasoju et al.) which carries out an analysis within the fast delivery services sector, which proposes an analysis model using Random Forest and XGBoost to forecast delivery times, which will allow to generate delivery strategies based on the characteristics of the users, on the one hand there are the highly satisfied who could accept a flexible delivery window, however, those who are dissatisfied could present a prioritization to improve their perception of the service. As can be seen, both articles seek to use prediction strategies that allow the company to anticipate adverse results and take improvement strategies.

At the same time, the techniques of this Research Topic have also been applied to strategies within the agricultural sector, as is the case of the article “*The evaluation of performance for agroecological greenhouse tomato strategies by the CRITIC-OWA model*” (Brotons-Martínez and Cámara-Zapata) which presents a new technique combining CRITIC (CRiteria Importance Through Inter-criteria Correlation) method with Ordered Weighted Averaging (OWA) and Induced Probabilistic OWA (IPOWA) operators. With this new methodology, it was possible to generate an analysis that balances different economic, environmental, and social elements, allowing visualization of the problem more holistically and assigning different weights to each of the study variables. This allows improving the process of generating policies and strategies at the corporate level. A sixth article that integrates this compilation is “*Trust by design in AI-augmented procurement systems: the roles of explainability, governance, and human oversight*” (Mejri and Skhiri) which analyzes the theme of negotiation between buyer and seller, analyzing the confidence that the use of artificial intelligence systems has within the negotiation processes, within the findings it is found that although there is a positive perception of the subject, it must be worked with care and specify the limitations that these could have within the negotiation process.

The seventh article within this compilation is entitled “*A systematic literature review on the use of multi-criteria decision-making methods for small and medium-sized enterprises innovation assessment*” (Rodríguez-Carrillo et al.) which presents a systematic review of the literature that explains how multi-criteria methodologies for decision-making in innovation within small and medium-sized enterprises have been applied. Among the main findings, they find that the research can be divided into four relevant topics: Innovation Capacity and Business Strategies, Open Innovation, Evaluation and Management, Technological and Digital Innovation, and Green Innovation and Sustainability. This allows us to visualize the actions that can be carried out within SMEs to help them improve the quality of their work, improve their indicators, and increase their lifetime within the markets.

As can be seen with all the previous cases, we can conclude that the use of soft computing and artificial intelligence techniques allows us to improve decision-making and analysis processes in management and engineering, which allows us to generate better results and improve indicators within organizations, so these methodologies must continue to be studied and applied in different sectors to have their applicability in different areas that improve the organizational processes.
